# Molecular Mechanisms Driving the Formation of Brain Metastases

**DOI:** 10.3390/cancers14194963

**Published:** 2022-10-10

**Authors:** Bethany K. Campbell, Zijie Gao, Niall M. Corcoran, Stanley S. Stylli, Christopher M. Hovens

**Affiliations:** 1 Department of Surgery, University of Melbourne, Parkville, VIC 3050, Australia or; 2 University of Melbourne Centre for Cancer Research, Victorian Comprehensive Cancer Centre, Parkville, VIC 3050, Australia; 3 Department of Urology, Royal Melbourne Hospital, Parkville, VIC 3050, Australia; 4 Department of Urology, Western Health, Footscray, VIC 3011, Australia; 5 Victorian Comprehensive Cancer Centre, Melbourne, VIC 3000, Australia; 6 Department of Neurosurgery, The Royal Melbourne Hospital, Parkville, VIC 3050, Australia

**Keywords:** brain metastases, genomics, secondary brain tumor, primary tumor, metastasis

## Abstract

**Simple Summary:**

Brain metastases are the most common brain tumor in adults and are associated with poor prognosis. The propensity of different solid tumors to metastasize varies greatly, with lung, breast, and melanoma primary tumors commonly leading to brain metastases, while other primaries such as prostate rarely metastasize to the brain. The molecular mechanisms that predispose and facilitate brain metastasis development are poorly understood. In this review, we present the current data on the genomic landscape of brain metastases that arise from various primary cancers and also outline potential molecular mechanisms that drive the formation of distant metastases in the brain.

**Abstract:**

Targeted therapies for cancers have improved primary tumor response rates, but concomitantly, brain metastases (BM) have become the most common brain tumors in adults and are associated with a dismal prognosis of generally less than 6 months, irrespective of the primary cancer type. They most commonly occur in patients with primary breast, lung, or melanoma histologies; however, they also appear in patients with other primary cancers including, but not limited to, prostate cancer, colorectal cancer, and renal cell carcinoma. Historically, molecular biomarkers have normally been identified from primary tumor resections. However, clinically informative genomic alterations can occur during BM development and these potentially actionable alterations are not always detected in the primary tumor leading to missed opportunities for effective targeted therapy. The molecular mechanisms that facilitate and drive metastasis to the brain are poorly understood. Identifying the differences between the brain and other extracranial sties of metastasis, and between primary tumors and BM, is essential to improving our understanding of BM development and ultimately patient management and survival. In this review, we present the current data on the genomic landscape of BM from various primary cancers which metastasize to the brain and outline potential mechanisms which may play a role in promoting the formation of the distant metastases in the brain.

## 1. Epidemiology of Brain Metastases

Brain metastases are a major contributor to cancer morbidity and mortality. Brain metastases are the most common cause of intracranial neoplasms in adults, arising 10 times more frequently than de novo brain cancers [1,2]. Additionally, they are the primary cause of neurologic complications stemming from systemic cancers [3]. The SEER database reported that between 2010–2013, 2% of all patients with a new cancer diagnosis presented with a brain metastatic disease [4]. It is estimated that between 14% and 20% of cancer patients will develop a metastatic brain tumor at some point during their treatment. This means that of the 1.7 million new cancer diagnoses per year in the United States, between 238,000 and 340,000 can expect to develop brain metastasis during their disease course. Recent trends have indicated that the incidence of brain metastases is increasing. Several factors may be contributing to this phenomenon, including longer cancer survival due to improved systemic therapies and increased surveillance modalities, as well as an increased populational cancer burden as a result of an aging population. A brain metastasis is an indication of poor prognosis, with short overall survival, progression-free survival, and time to neurological deterioration [5]. Historically, patients with brain metastasis have a median overall survival of <6 months, irrespective of the primary cancer type [6,7]. The 5-year survival rate for patients with brain metastases is under 2% [1].

## 2. Treatment of Brain Metastases

### 2.1. Current Standard of Care

Conventional treatments of brain metastasis include surgical removal, whole brain radiation therapy, and chemotherapy, and are traditionally performed for palliative reasons. More recently, advancements in systemic medical oncology, surgical techniques, and technology and radiation therapy have provided alternative treatment strategies [8,9,10]. Treatment decisions involve a multidisciplinary team of clinicians and are based on a collection of consensus guidelines from several organizations including the Congress of Neurological Surgeons [11,12,13,14] and the consortium of the American Society of Clinical Oncology, the Society for Neuro-Oncology, and the American Society for Radiation Oncology [3], which consider patient factors such as the number of metastases, performance score, and location of metastasis. Treatment of brain metastases is performed with the aim to achieve local control of the metastatic lesion, improve quality of life, and prevent death from neurological disease [15].

### 2.2. Emerging Treatment Strategies

In general, systemic therapies are often obstructed to acting at the site of a brain metastasis by the blood–brain barrier and, as such, it is unsurprising that chemotherapies have shown disappointing efficacy. Developments of new small molecule therapies and recent advances in our understanding of cancer biology means we are beginning to see targeted therapies show promising results in patients with brain metastases, especially when combined with conventional therapy strategies [8,9,10,16]. A time-series based meta-analysis reported that immune checkpoint inhibitors may provide clinical benefit. The analysis showed the best survival outcome with anti-cytotoxic T-lymphocyte antigen 4 (CTLA-4) and programmed cell death protein 1/ligand 1 (PD-1/PDL1)-based treatment, with this result strongest in patients with a melanoma primary tumor [17]. Targeted therapies that tackle molecular drivers in the cell signaling pathway have progressed rapidly in the treatment of cancer, and they are an active area of research for the treatment of brain metastases. A retrospective study by Yomo et al. reported that treating brain metastases arising from epidermal growth factor receptor (EGFR)-mutant lung cancer with EGFR tyrosine kinase inhibitors resulted in a 1-year survival rate of 74% and a 2-year survival rate of 52% [18]. Similarly, the use of tyrosine kinase inhibitors in combination with stereotactic radiosurgery in human epidermal growth factor receptor 2 (HER-2)-amplified breast cancer brain metastases patients reported higher rates of complete response, compared to stereotactic radiosurgery (SRS) alone [19]. In a second study in the sample population, concurrent SRS and tyrosine kinase inhibition was associated with reduced local failure compared to nonconcurrent therapy [19]. Despite these improvements in longevity, the quality of life for these patients remains poor, due to neurologic and cognitive impairment [4,20]. 

## 3. Factors Influencing Brain Organotropism 

Primary tumors from different organs exhibit a preference in the organs to which they metastasize. This phenomenon is known as ‘organotropism’. Organotropism is thought to be regulated by a variety of factors, including circulatory and anatomical proximity, metastatic niche, and tumor cell intrinsic factors. The interaction between the cell-intrinsic properties and the metastatic niche microenvironment is known as the ‘seed and soil’ hypothesis, originally proposed by Stephen Paget in 1889. 

There is a marked difference in the propensity of solid organ tumors which give rise to brain metastases. The three most common primary tumors associated with brain metastases are lung (20–56%), breast (5–20%), and melanoma (7–16%). Other cancers which commonly metastasize to the brain include colorectal, ovarian, and kidney. In contrast, prostate, head, and neck and non-melanoma skin cancers rarely metastasize to the brain [4,21,22,23,24]. 

### 3.1. Formation of the Pre-Metastatic Niche

Metastasis to the brain, like any organ, requires complex communication networks between the invading cell and resident cells of the metastasis location. Before arrival at the site of metastasis, tumor cells can modulate the microenvironment to be more conducive of survival and outgrowth. This hypothesis is known as the formation of the pre-metastatic niche. Cancer cell-derived extracellular vesicles have been shown to be one of the key players in the formation of the pre-metastatic niche [25,26]. A study by Hoshino et al. profiled the exosomal integrins and found unique expression patterns which correlated to organ-specific colonization of the tumor cells from which they were derived [26]. Furthermore, they found that tumor-secreted exosomes were sufficient to direct organ-specific metastasis. Beyond directing the tumor cell to the site of metastasis, exosomes are filled with a millennium of biomolecules which can prepare the metastatic niche to support tumor growth. For example, Rodrigues et al. have shown that biomolecules within extracellular vesicles promote cancer cell colonization by stimulating endothelial branching and the establishment of a pro-inflammatory vascular niche to support metastatic outgrowth [27]. 

### 3.2. Transendothelial Migration across the Blood–Brain Barrier

Perhaps the most unique barrier preventing metastasis of the brain is the existence of the blood–brain barrier (BBB), a continuous endothelium which tightly regulates access to the brain. In order for cells to metastasize to the brain, they must cross this barrier, which can be achieved by disruption of the structural integrity of the BBB. Brain metastatic cells have been shown to produce cathepsin S, which proteolyzes the junctional adhesion molecules, thus allowing the passage of tumor cells [28]. In small cell lung cancer (SCLC), Li et al. reported that significantly higher sera levels of placental growth factor (PLGF) are observed in patients with brain metastases compared to those without [29]. An in vitro model using 3 SCLC cell lines showed the highest PLGF expression in the cell line obtained from a brain metastatic site. They also showed this cell line had the highest trans-endothelial migration ability, which was reduced by antibody-mediated PLGF neutralization. A collection of studies has demonstrated that tumor cell-derived exosomes contain biomolecules, such as microRNAs and long non-coding RNAs, which can impact the BBB permeability [30,31,32]. For example, exosomes have been shown to contain microRNAs, such as *miR-181c* and *miR-105*, which lead to disruption of cellular junctions in the BBB [31,32]. Interestingly, one study reported that sera from breast cancer patients with brain metastasis have been shown to have higher expression of *miR-181c* in extracellular vesicles compared to those patients without brain metastases [31]. The *miR-509* has also been described to impact BBB permeability and invasion by modulating the Rho-TNFα signaling network. A study by Xing et al. reported decreased expression of *miR-509* in brain metastatic lesions from breast primaries compared to the primary. This was further validated by an in vivo study that demonstrated *miR-509* suppresses brain metastasis formation [33].

### 3.3. Brain-Specific Cell Types; A Support Network

Once tumor cells have passed the BBB, the brain microenvironment presents further challenges to their growth. The brain consists of neurons and glial cells which can sustain the tumor cells by hijacking this support system. For example, the secretion of growth factors and cytokines by astrocytes can stimulate tumor cell growth, including astrocyte-derived interleukin 6 (IL-6), transforming growth factor-beta (TGFβ) and insulin-like growth factor 1 (IFG-1) which have been shown to increase tumor cell proliferation [34,35]. Astrocytes can also reprogram the genetics of invading tumor cells to support metastasis. One example of this is via astrocyte-derived exosomes, which are enriched with *miR19a*, a microRNA which targets phosphatase and tensin homolog (PTEN). Tumor cells exposed to miR19a have decreased expression of PTEN, a key tumor suppressor, which primes the cells for metastatic outgrowth [36].

Similar to primary tumors, tissue-resident immune cells, particularly macrophages, play a major role in the formation of the pre-metastatic niche and promotion of tumor cell colonization and proliferation. The majority of tumor-associated macrophages found within brain metastases are brain-resident microglia, which have undergone a change from a resting ramified phenotype towards an activated amoeboid morphology with multiple small protrusions [37,38,39,40]. These microglia can make up anywhere from a few cells to 50% of the lesion cellular content [39]. The presence of microglia is not restricted to the necrotic regions of the tumor. They are also found in the border zone between tumor and neighboring brain tissue, as well as in the infiltration zone of the tumor [39].

Microglia have been shown to be able to suppress local immunity and thereby enhance invasion and colonization of brain tissue by tumor cells to promote formation of brain metastases. A study by Pukrop et al. utilized in vitro co-culture with breast cancer cells and microglia in an organotropic brain slice culture to demonstrate that microglia promote cancer cell invasion and colonization of brain tissue [39]. Microglia were shown to facilitate the transport of both single invading cells and small cohorts of cells. These functions were inhibited by treatment with bisphosphate clodronate, an inhibitor of the microglia. This microglia-mediated invasion was dependent on Wnt/B-catenin and PI3K signaling pathways [39]. Furthermore, Chuang et al. demonstrated that microglia support the invasion of cancer cells, but not benign epithelial MDCK cells [41]. These data are supported by in vivo studies which have shown that activated microglia are recruited to the tumor–brain interface [42]. Additionally, Qiao et al. reported that depletion of tumor-associated macrophages, including microglia, reduced the total number and mean size of brain metastases [43]. Together, these data support a critical role for microglia in invasion and colonization of brain tissue by epithelial cancer cells.

### 3.4. Adaptation to the Brain Microenvironment 

Tumor cells invading the brain need to propagate under specific metabolic conditions, in particular conditions of hypoxia and glucose shortage [44,45,46]. Several studies have shown that invading tumor cells can alter their nutrient metabolic requirements to reduce dependence on glucose and exploit locally available nutrients such as acetate, glutamine, and gamma-aminobutyric acid (GABA) [47,48]. Fatty acid synthesis pathways have also been shown to be altered in brain metastatic cells. Jin et al. reported a strong correlation between brain metastatic potential and expression of a lipid-synthesis signature in a study of 500 human cells lines [49]. They observed increased levels of cholesterol species and decreased triacylglycerol species in brain metastatic cells compared to non-brain metastatic cells. A second study by Farraro et al. demonstrated that fatty acid synthesis pathways are upregulated in breast cancer cells which metastasize to the brain [50]. Together, these studies demonstrate adaptation to the nutrient environment of the brain, promoting survival. 

## 4. Genomic Alterations Observed in Brain Metastases and Primary Tumors

Whether brain metastases were molecularly similar to the primary tumors from which they arise was largely unknown until recently with the evolution of technology such as next-generation sequencing. Numerous studies have attempted to compare the molecular landscape of BM and primary tumors with several studies that have investigated genomic alterations and the potential drivers in BM presented in Table 1. 

In a study involving the whole-exome sequencing of 86 matched primary tumors and brain metastases using a pan-cancer approach, it has been demonstrated that even though there is common ancestor between paired primary tumors and brain metastasis pairs, a defined evolutionary pattern occurs at each metastatic site [64]. They found that in 53% of cases, potentially clinically informative alterations were present in the brain metastases that were not detected in the matched-primary tumor samples. The TARGET database of genes with somatic alterations that have therapeutic or prognostic implications was utilized to organize the analysis of the paired samples and, out of 95,431 gene alterations, 330 genes satisfied the TARGET criteria of being clinically informative [65]. Alterations potentially predicting sensitivity to cyclin-dependent kinase (CDK) inhibitors were common with 71 alterations in 48 cases occurring in 10/11 evaluated genes. From the 71 alterations, 44 were shared between primary tumor–brain metastases, seven were only present in the primary samples, and 20 were detected only in the brain metastasis sample. The most frequently altered gene was *CDKN2A*, which included 17 events in total (including homozygous deletions in 3/8 colorectal cancer cases which were only present in the brain metastasis). *MCL1* amplifications (sensitive to CDK inhibitors) in 5/15 events were detected in the brain metastasis samples. PI3K-AKT-mTOR pathway-related mutations (43) were detected in 37 cases in 10 of the 15 evaluated genes, with 24/43 being shared, 5/43 detected in the primary cancers, and 14 detected in the brain metastases. The occurrence of actionable alterations in these genes was observed as follows: breast cancers (9/21 cases; 6/9 shared primary–brain metastases) and lung adenocarcinoma (12/29 cases; 8/12 shared primary–brain metastases). It was also observed that anatomically distinct brain metastases in patients were more closely related to each other than the corresponding primary cancer, while also harboring identical relevant clinical information. This was observed in a patient with an HER2-amplified salivary gland ductal carcinoma who developed a brain metastasis with clinically informative amplifications (*MET*, *CDK6*, *CCNE1*, *MYC*, and *AKT2*) that were not detected in the primary tumor. However, after a 10-month period post-irradiation, the patient developed a new brain metastasis in the parietal lobe with identical amplifications.

A study by Dono et al. performed next-generation sequencing on a retrospective cohort of 144 BM patients by testing for genomic alterations on a set of 315 genes in a pan-cancer study [55]. In a comparison between the BM and primary tumors, the following genes were mutated in BM with increasing frequency: *TP53*, *ATR*, and *APC* (lung adenocarcinoma); *ARID1A* and *FGF10* (lung small-cell); *PIK3CG*, *NOTCH3*, and *TET2* (lung squamous); *CDKN2A/B, PTEN, RUNX1T1, AXL*, and *FLT4* (melanoma); *ERBB2*, *BRCA2*, and *AXL1* (breast carcinoma); and *ATM*, *AR*, *CDKN2A/B*, *TERT*, and *TSC1* (renal clear-cell carcinoma). In addition, they determined that breast cancer BM patients with *ERBB2*, *CDK12,* or *TP53* mutations and lung adenocarcinoma BM patients with *CREBBP*, *GPR124,* or *SPTA1* mutations have worse prognoses. Shih et al. performed whole-exome sequencing of 73 BM–lung adenocarcinoma cases, and by identifying genes with more frequent copy-number alterations compared to a cohort of 503 primary lung adenocarcinomas, there were significantly higher amplifications frequencies of the BM for *MYC* (12% vs. 6%), *YAP1* (7% vs. 0.8%), *MMP13* (10% vs. 0.6%), and more deletions in *CDKN2A/B* (27% vs. 13%) [53]. An independent cohort of 105 patients was also utilized to confirm the amplification frequencies of *MYC*, *YAP1,* and *MMP13*.

A small study by Aljohani et al., involving whole genome sequencing of normal lung, primary tumor and the corresponding BM from 5 patients with progressive non-small cell lung cancer (NSCLC), revealed that primary tumors were associated with mutations in cell adhesion and motility, whereas BM acquired mutations in adaptive, cytoprotective genes such as *KEAP-1*, *NRF2*, and *P300* [56]. An important observation by these authors was that they were able to detect these mutations in circulating tumor cells (CTCs) from the peripheral blood of 10 patients with either metastatic melanoma, breast, or colon cancer, suggesting that the Keap1-Nrf2-ARE cell survival pathway provides a survival advantage for these cells allowing them to form metastatic tumors in distant organs. Li et al. sequenced 7 triple samples of primary NSCLC tumors, adjacent normal tissue, and the corresponding BM [54]. The WES method detected more than 20,000 exons to provide a clearer representation of the differences between the samples. The two genes, *FAM129C* and *ADAMTS*, demonstrated a stronger correlation with BM. In addition, they observed copy number deletions with *SAMD2* and *SMAD4*, which are part of the TGFβ signaling pathway that is involved with BM. They also observed *TP53* and *EGFR* mutations in both primary and BM tissue, as also reported by previous studies.

A Nanostring nCounter PanCancer Immune profiling panel comprised of 770 immune-related genes was utilized by Song et al. to characterize the population differences between primary NSCLC tumors and brain metastases [66]. Fifty-four genes were significantly differentially expressed between primary and brain metastatic tumors and tumors which contained mutated *EGFR*, as well as diverse immune-related pathways being upregulated in the BM. Thirty-six genes were significantly upregulated in the primary lung cancer and eighteen in the corresponding BM. Genetic markers of T cells and B cells (*CD3E* and *CD79A*) were upregulated in the primary tumor, whereas M2 macrophage/microglia-related (*CD163*) and natural killer-cell-related (*CD56*) genes were upregulated in the BM, alongside anti-inflammatory markers, toll interacting protein (*TOLLIP),* and human leukocyte antigen G (*HLA-G)*. *EGFR* mutation in the primary cancer was associated with a lower PD-L1 expression, T-cell infiltration, and a tumor mutation burden. Jiang et al. undertook a comprehensive whole-exome sequencing analysis of primary lung adenocarcinoma, blood, and lung or brain metastases from 26 patients [67]. They discovered that common driver mutations, including *TP53* and *EGFR*, were consistent between paired primary and metastatic tumors, although the liver metastases demonstrated a similar mutational landscape than the BM samples when paired with the primary cancer, suggesting that actionable mutations identified from a single biopsy taken from the primary cancer may not represent in the mutations observed in the BM. This indicated that distinct mutational and evolutionary trajectories are involved in the metastases to different organ sites from the same primary tumor.

Sanus et al. performed an integrated genomic and transcriptomic unmatched analysis of 36 BMs from multiple primary tumor types (breast, lung, melanoma, and esophageal) which discovered novel candidates with potential roles in BM development, including significantly mutated genes *DSC2*, *ST7*, *PIK3R1*, and *SMC5*, in addition to DNA repair, ERBB–HER signaling, axon guidance, and protein kinase-A signaling pathways [68]. In addition, a mutational signature analysis was applied to successfully identify the primary cancer for two BMs with unknown origins present in the cohort, with actionable genomic alterations also identified in 86% (31/36) of the BM samples supporting a genotype–drug efficacy relationship. High expression levels of the growth factor receptor *HER3* were detected in the BMs, even though the ligand neuregulin I was low in the tumor cells. The brain microenvironment is rich in the ligand, which allows the tumor cells to survive and proliferate into a BM, also highlighting the importance of the relationship between the brain microenvironment and the tumor cells. Although this was a retrospective study, the authors proposed that it was possible that this approach in a prospective study may have impacted on some of the cases if known at the time of resection, as 10 patients may have been eligible for a phase I trametinib (MEK-inhibitor) trial due to NRAS- or KRAS-mutant cancers.

Fukumara et al. performed a multiomic profiling-based study, where they undertook genomic, transcriptional, and proteomic profiling using whole-exome sequencing, mRNA-seq, and reverse phase protein array analysis on a cohort of lung (14 patients), breast (14 patients), and renal cell carcinomas (7 patients) that were primary and matched BM or extracranial metastatic (EM) cancers [52]. The clinical specimens were surgically resected normal or tumor tissues and patient-matched white blood cells. While they were not able to identify specific genomic alterations associated with BM in this cohort, there were correlations with impaired cellular immunity, upregulated oxidative phosphorylation (OXPHOS), and canonical oncogenic signaling pathways (including phosphoinositide 3-kinase (PI3K) signaling) across multiple histologies. However, they did observe mutations and copy number alterations distinguishing individual BMs from their patient-matched P/EM samples. The mutational profiles were largely reflective of the cancer type, falling within established rates for the three cancers and with no significant difference between primary/EM cancers versus BM status.

Despite the absence of genomic alterations associated with BM in the study by Fukumara, identifiable gene mutations in BM generally provide insights into shared gene alterations which are common across brain metastases [52]. As BM generally occur late in a patient’s disease course for the primary tumor, resistance to the initial targeted therapies based on the primary tumor or loss of previously identified biomarkers result in the initial therapies being ineffective. Therefore, molecular signatures are frequently observed to be different between the primary cancer and the corresponding BM, as a result of clonal evolution during migration of the tumor cells, systemic treatments providing selective pressure, and differences in the local microenvironment. 

This is likely to lead to critical future therapeutic developments in the treatment of BM patients, accounting for varied clinical behavior that is observed between BM patients. Importantly, as the mutational signature can evolve over time in response to treatment, it will be vital to determine the changes in the genetics of BMs, as this may lead to modifications in the therapeutic protocol and the overall clinical management of the patients. The functions of significant genes in BM and the current available targeted therapies are outlined in Table 2.

However, BM-targeted therapies based on molecular biomarkers/signatures will also depend largely on the experience of the treating clinicians, who may have little experience in determining the efficacy of the targeted therapy based on current knowledge of the therapy with other diseases, as well as the ability to cross the blood–brain barrier. Thus, some institutions may rely on a multidisciplinary approach through ‘Molecular Tumor Boards’, with input from multiple specialties, which would enhance the clinical care based on a genomics approach. As ‘Molecular Tumor Boards’ are adopted across multiple hospital sites, knowledge on how to effectively implement targeted therapies based on the molecular differences between BM and their primary cancers will result in a better prognosis for BM patients.

## 5. Monoclonal Verses Polyclonal Spread of Brain Metastases

Brain metastases are seeded by highly evolved primary tumors in NSCLC cases. Brain metastases derive from late-arising tumor clones in primary NSCLC tumors. A study from Lee et al. looked at the relationship of somatic mutations present in primary tumors with matching somatic mutations in corresponding brain metastasis samples in 7 patients with NSCLC [69]. Interestingly, the metastatic samples sequenced in this study were sampled across a wide temporal spacing ranging from 3 to 24 months after the time point the primary tumors were sequenced with an average of 9 months. Nearly 70% of the mutations detected in the metastatic samples were present in the primary tumors, implying that the metastatic seeding events occurred late in the primary tumor evolutionary cycle. To determine whether this pattern of seeding was observed in distinct cohorts, Lee et al. conducted a similar analysis using somatic WES data derived from 35 primary–brain metastasis samples from the Brastianos cohort of NSCLC tumors [64]. Similar to their original findings, they observed that the rate of shared mutations between primaries and brain metastases in the distinct cohort was validated with approximately 70% of somatic exonic variants shared between the tumor locations, suggesting that brain metastases derive from late-evolving subclones in primary lung tumors. 

This finding is similar to that reported in an earlier larger study of brain metastatic lung adenocarcinoma series where 73 brain metastatic samples and matching primary tumor tissue from 58 of these cases were whole exome-sequenced to high depth [53]. They were able to assess the significance and evolutionary timing of candidate somatic driver events in either of the primary or metastatic samples, whether private and assumed to have occurred after the divergence of the metastatic and primary tumor lineages. Variants that were shared by the primary tumor sample and brain metastasis were assumed to have occurred in an ancestral population that preceded their divergence. This analysis also confirmed that metastatic subclones developed late in the tumor evolution of the primary cancer, with the vast majority of somatic variants clonally shared between the two tumors. Of interest, deletions of *CDKN2A/B* had a higher propensity to be shared between the metastatic and primary tumor lineages, suggesting that loss of this region was positively selected and had a significant role in advancing the metastatic potential of primary tumors. 

Fukumura et al. performed genomic, transcriptional, and proteomic profiling in a cohort of 35 patients comprising 14 lung, 14 breast, and 7 renal cell carcinomas, consisting of both BMs and patient-matched primary or extracranial metastatic tissues [52]. Two distinct brain metastatic foci were isolated from two different lung cancer patients, providing an opportunity to observe inter-metastatic heterogeneity in these patients. The tissues were from fresh frozen samples and matching primary tumors were available from 33 of the patients in the cohort. However, it is not clear in all cases whether the primary and brain metastatic tissues were sampled asynchronously, permitting observance of evolutionary changes across time, or whether some samples were isolated at the same time. An analysis of the whole-exome sequencing data was used to explore the landscape of point mutations and copy number changes in the paired brain metastatic and primary tumor specimens. The overall burden of SNV mutations and CNV profiles reflected the patterns typically observed in the three primary tumor types utilized in this study. Even though the overall mutation burden was not significantly different between the respective paired brain metastases and primary tumors, there were in some cases large clusters of mutations private to either the brain metastases or the matching primary tumor specimens. This would suggest that at least in some of these cases the seeding of the brain metastatic clones occurred early in the primary tumor evolution and was not likely to be a late event, as has been observed in the other studies previously mentioned. An analysis of the mutations private to the seeding clones would likely be informative as to whether there would exist any driver mutations common across the three different tumor types. Additionally, this study did also not report on the clonality status of either the brain metastases or the primary tumors. Hence, it is not possible to confirm whether the brain metastases analyzed in this study had the predominant monoclonal status, as has been reported in other studies, and which would appear to demarcate brain metastases from other sites of metastasis where polyclonal status of metastases is more common [70]. 

## 6. Are Brain Metastases Seeded Directly from Primary Tumors or from Other Extracranial Metastases?

One of the outstanding questions relating to the seeding of brain metastases is from which particular tumor site do they most likely seed from. That is, whether they seed directly from subclones arising in the primary tumor site or whether they are formed from secondary waves of dissemination from extracranial sites of metastasis in a hierarchy of spread. To date, no systematic study addressing this question has been reported. There is only anecdotal evidence that brain metastases and other extracranial metastases in the same patients have very similar somatic variants and extent of tumor evolution, suggesting that they likely seed at the same time but in what order or whether the disseminations are independent is still preliminary [52]. Vergara et al. have conducted one of the most thorough analyses performed to date of the evolutionary relationship between primary and distant metastatic lesions in melanoma patients [71]. They reported in detail on the phylogenetic relationships between multiple lesions in three patients with concomitant brain lesions and other extracranial metastases. Interestingly, this analysis unequivocally revealed that each of the brain metastases were seeded from precursor clones that could be directly linked back to the primary tumors and not from clones derived from other distant metastases [71]. This would suggest that the potential for brain metastasis formation is hardwired in some primary tumors rather than formed by a stepwise progression from other distant metastases as a last site of dissemination. However, a definitive answer to this question awaits more comprehensive analyses.

## 7. Conclusions

The development of brain metastases is the final harbinger of the terminal phase of the disease for many cancer patients. The incidence of brain metastases is paradoxically rising as targeted therapies improve tumor control in the extracranial environment. What drives brain metastasis formation is largely underexplored and therapies targeting this process are still experimental. Recent studies have helped to elucidate the landscape of genomic alterations enriched in brain metastases, as well as the mechanisms that potentially drive this process. This knowledge could help inform future treatment strategies, as well as provide possible early-stage screening of primary tumors likely to spread to the brain. 

## Figures and Tables

**Table 1:** Genomic alterations and potential drivers in BM from different primary cancers.

Study	Sequencing Method	Cohort	Sample Type	Pathology	Genes Amplified, Overexpressed, Activated, or Gain of Function	Genes Loss, Suppressed, Inactivated, or Loss of Function	Genes Mutated
Z. Song et al. [51]	Next-Gen Panel sequencing	32 BMs and 25 primaries (24 matched samples plus 8 with only BMs and 1 with only primary)	FFPE	Lung	*EGFR*	*TP53* (50 versus 40%), *ZFHX3* (28 versus 40%)	*RBM10* * (6 versus 28%), *ARID1B*, *MLL3*, *FAT2*
K. Fukumura et al. [52]	WES, RNA-seq	14 matched samples	Fresh-frozen and FFPE	Lung	*EGFR* (4/14 versus 1/14)	*TP53*, *ATM* (4/14 versus 3/14), *LRP1B* (9/14 versus 7/14), *PTPRD* (7/14 versus 6/14), *FAT1*(6/14 versus 5/14)	*MLL2* (3/14 versus 2/14), *MLL3* (6/14 versus 4/14)
D.J.H. Shih et al. [53]	WES	Unmatched 73 BM and 513 primaries LUAD sequenced by TCGA	Fresh-frozen and FFPE	LUAD	*MYC* * (12 versus 6%), *YAP1* * (7 versus 0.8%), *MMP13* * (10 versus 0.6%), *KEAP1*, *EGFR*, *TERT*	*CDKN2A/B* * (27 versus 13%), *SKT11*, *TP53*	*KRAS*
L. Li et al. [54]	WES	7 matched samples with BM and primaries	FFPE	LUAD	*EGFR*, *ADAMTSs*, *NKX2-1*, *DDR2*, *MAPK3*, *MCL1*, *MYC*	*TP53*, *SMAD2*, *SMAD4*	*FAM129C*, *NOTCH1*, *EPHA5*, *ATP2B1*
A. Dono et al. [55]	Next-Gen Panel sequencing	60 unmatched samples	Unspecified	LUAD	--	TP53 *, APC *, ATR *	--
A. Dono et al. [55]	Next-Gen Panel sequencing	10 unmatched samples	Unspecified	SCLC	*FGF10* *	--	ARID1A*
H.M. Aljohani et al. [56]	WGS	5 matched BM and primary	Unspecified	NSCLC	*KEAP1*, *Nrf2* *, *EP300* * (4/5 versus 0/5)	--	--
L. Liao et al. [57]	WES, Next-Gen Panel sequencing	6 matched samples with BM and primaries	FFPE	NSCLC	*EGFR*	*TP53*, *ATXN1*, *LRP1B*, *MSH2*, *FANCD2*	*NOTCH2/NOTCH2NL*
K. Fukumura et al. [52]	WES, RNA-seq	14 matched samples	Fresh-frozen and FFPE	Breast	*HER2* (43 versus 29%), *CDK12* (43 versus 29%)	*TP53*	--
M. B. Siegel et al. [58]	WES, RNA-seq	16 matched samples	Fresh-frozen and FFPE	Breast	*ANGPT1*, *LYN*, *SDC2*, *SHC1*, *GDNF*, *TERT.* Basal-like (TN) specific: *CCNE1*, *CUL1*, *CDK5*, *RBBP4*, *HDAC1*, *BCAN*	*FAS*, *PIK3R1*, *AURKB*, *TP53*. Basal-like (TN) specific: *RAD51*	*ESR1*
M. Tyran et al. [59]	Whole genome array comparative genomic hybridization	14 matched samples	Fresh-frozen	Breast	*CCND1*, *MYC*, *HER2*, *PIK3CA*	*TP53*, *RB1*	*MLL2*, *MLL3*, *COL6A3*, *MDM4*
A. Dono et al. [55]	Next-Gen Panel sequencing	21 BMs and primary data sequenced by COSMIC and TCGA	Unspecified	Breast	*HER2* *, *ASXL1* *	*BRCA2* *	--
A. Dono et al. [55]	Next-Gen Panel sequencing	14 BMs and primary data sequenced by COSMIC and TCGA	Unspecified	Melanoma	*AXL* *, *FLT4* *	*CDKN2A*/*B* *, *PTEN* *	*RUNX1T1* *
G. Chen et al. [60]	Whole genome wide expression profiling	16 matched melanoma BM and extracranial metastases	Fresh-frozen and FFPE	Melanoma	*TBX2*, *SGK3*, *SGSM2*, *ELOVL2*	CDKN2A *, *PTEN*	--
Z. Hu et al. [61]	WES	10 matched CRC BM and primary	FFPE	CRC	*PIK3CA*, *GNAS*, *SRC*, *FXR1*, *MUC4*, *GPC6*, *MECOM, HTR2A* *	--	--
J. Sun et al. [62]	WES, WGS	19 matched CRC BM and primary	FFPE	CRC	--	*RAD51*, *PAXIP1*, *XRCC4*	*MUC19*, *SCN7A*, *SCN5A*, *SCN2A*, *IKZF1*, *PDZRN4*
K. Fukumura et al. [52]	WES, RNA-seq	6 matched samples	Fresh-frozen and FFPE	RCC	*PIK3CB*	*BAP1*, *VHL*	*TP53*
Y. J. Choi et al. [63]	WES	1 matched BM and primary	FFPE	Peritoneal	--	*RAP1GDS1*, *TET2*, *IL2*	--
Y. J. Choi et al. [63]	WES	1 matched BM and primary	FFPE	Ovarian	--	*RAP1GDS1*, *TET2*, *IL2*	--

* Represents gene expression that is significantly altered in BM compared to primary/EM.

**Table 2:** Functions of significant genes in BM and available targeted therapies.

Gene	Gene Function	Types of BM	Targeted Therapy
*EGFR*	Epithelial growth factor receptor, cell growth and proliferation	Lung	EGFR inhibitors: Erlotinib, Gefitinib, etc.
*TP53*	Tumor suppressor	Lung, breast, CRC	--
*APC*	Tumor suppressor	LUAD, CRC	--
*PTEN*	Tumor suppressor	Lung, breast, melanoma	--
*ATR*	Tumor suppressor	LUAD	--
*RB1*	Tumor suppressor, cell cycle regulator	Breast	--
*MSH2*	DNA repair, tumor suppressor	Lung	
*RBM10*	RNA-binding Motifs (RBM) which belong to a large family of RNA-binding proteins, post-translational processing, probably mRNA splicing	Lung	--
*ZFHX3*	Tumor suppressor, transcription factor	Lung	--
*ARID1A/ARID1B*	Tumor suppressor, member of SWI/SNF chromatin remodeling complex	Lung	--
*FAT1/FAT2*	FAT atypical cadherin 1, FAT atypical cadherin 2, cell proliferation, migration, and invasion	Lung	Potential target for therapy
*MLL3*	Lysine methyltransferase, chromatin-regulating gene	Lung	--
*MLL2*	Lysine methyltransferase, chromatin-regulating gene	Lung	--
*ATM*	Serine/threonine protein kinase, DNA repair, cell cycle checkpoint	Lung	Potential targeted therapy: PARP inhibitor such as Rucaparib
*LRP1B*	Tumor suppressor, member of low-density lipoprotein receptor family	Lung	--
*ATXN1*	Chromatin-binding factor that represses Notch signaling	Lung	--
*PTPRD*	Protein tyrosine phosphatases receptor D, tumor suppressor	Lung	--
*MYC*	Cell progression, apoptosis, and cellular transformation in EGFR pathway	LUAD, breast	--
*YAP1*	Regulatory factor in the Hippo signaling pathway that regulate cell proliferation, death, and migration	LUAD	--
*MMP13*	Matrix degradation, cell invasion	LUAD	--
*CDKN2A/B*	Tumor suppressor, cell cycle checkpoint	LUAD, melanoma	CD4/6 inhibitors
*KRAS*	Cell proliferation	LUAD	KRAS inhibitors
*SKT11*	Tumor suppressor, serine/threonine-protein kinases	LUAD	Potential target of Bemcentinib, Everolimus, Talazoparib
*KEAP1, Nrf-2, EP300*	Involved in KEAP-Nrf2-ARE pathway, cell survival signaling under oxidative stress	LUAD, NSCLC	--
*CREBBP*	Transcriptional factor	LUAD	--
*FAM129C*	Niban apoptosis regulator 3, unclear function	LUAD	--
*DDR2*	Receptor tyrosine kinase, ECM remodeling by up-regulating collagenases	LUAD	TKIs such as Sorafenib
*MAPK3*	Mitogen-activated protein kinase	LUAD	EGFR inhibitor
*MCL1*	Anti-apoptotic protein, enhance survival by inhibiting apoptosis	LUAD	CDK inhibitors
*NOTCH1, NOTCH2/NOTCH2NL*	Notch receptor family in Notch signaling pathway	Lung	Notch inhibitors
*FANCD2*	Tumor suppressor, DNA repair,	NSCLC	--
*ADAMTS6/* *ADAMTS20*	ADAMTS family of zinc-dependent proteases, ECM remodeling	LUAD	--
*FGF10*	Fibroblast growth factor 10, cell proliferation and differentiation	SCLC	--
*NKX2-1*	Thyroid transcription factor 1	LUAD	--
*SMAD2/SMAD4*	Transcription regulator, involved in TGF-beta receptor signaling pathway	LUAD	--
*HER2/HER3*	Human epithelial growth factors, tyrosine kinase	Breast	HER2 inhibitors, tyrosine kinase inhibitors
*ANGPT1*	Vascular development, angiogenesis	Breast	Angioprotien-1 inhibitor: Trebananib
*LYN*	Protein tyrosine kinase, proto-oncogene	Breast	TKIs such as Bafetinib
*SDC2*	Cell surface proteoglycan	Breast	--
*SHC1*	Signaling adaptor, participate in angiogenesis and endothelial cells recruitment	Breast	--
*GDNF*	Glial cell line-derived neurotrophic factor, promotes survival and differentiation of neurons	Breast	--
*TERT*	Telomerase reverse transcriptase, telomere ends maintenance, cell senescence	Breast	--
*CUL1*	Cullin 1, enables ubiquitin protein ligase-binding activity	Breast	--
*PIK3R1*	Regulatory subunit of PIK3CA	Breast	
*FAS*	Critical in apoptosis cascade	Breast	
*AURKB*	Cell cycle pathway inhibitor	Breast	
*CDK5*	Cyclin-dependent kinase 5	Basal-like breast	--
*RBBP4*	Chromatin remodeling factor, cell cycle progression	Basal-like breast	
*HDAC1*	Histone deacetylase, transcriptional regulation, cell cycle progression	Basal-like breast	
*BCAN*	Proteoglycan, involved with formation of ECM in brain, promote growth and motility in brain tumor cells	Basal-like breast	
*RAD51*	Tumor suppressor, DNA repair	Basal-like breast	--
*ESR1*	Estrogen receptor 1	Luminal breast	Estrogen receptor antagonist/Hormone replacement agents
*CCND1/CCNE1*	Cyclin D1, E1 cell cycle progression	Breast	--
*FGFR4*	Fibroblast growth factor receptor 4	Breast	--
*PIK3CA/PIK3CB*	Protein tyrosine kinase	Breast, CRC	TKIs such as Idelalisib, Copanlisib
*ASXL1*	Chromatin-binding protein, transcription regulator	Breast	--
*COL6A3*	Encoding for one of the 3 alpha-3 chain of type VI collagen, important for ECM organization	Breast	--
*MDM4*	Regulator of TP53, cell apoptosis	Breast	--
*BRCA2*	Tumor suppressor	Breast	--
*RUNX1/RUNX1T1*	RUNX family transcription factor/RUNX family transcription factor co-Repressor	Melanoma	--
*AXL*	Receptor tyrosine kinase	Melanoma	TKIs such as Bemcentinib, Crizontibin
*FLT4*	Protein tyrosine kinase, lymphangiogenesis	Melanoma	TKIs such as Lenvatinib
*TBX2*	T box transcription factor, EMT, cell invasion	Melanoma	--
*SGK3*	Serine/threonine-protein kinase, cell growth, proliferation, and migration	Melanoma	--
*SGSM2*	GTPase activator that regulates melanogenesis	Melanoma	--
*ELOVL2*	Fatty acid elongation, process membrane lipids	CRC	
*GNAS*	Guanine nucleotide-binding protein	CRC	
*SRC*	Proto-oncogene, non-receptor tyrosine kinase	CRC	Dasatinib, Bosutinib, Tirbanibulin
*FXR1*	RNA-binding protein	CRC	
*MUC4/MUC19/MUC17*	Gel-forming mucin protein family, major constitutes of mucus	CRC	
*GPC6*	Proteoglycans	CRC	
*MECOM*	Transcriptional regulator, oncogene, cell proliferation and differentiation	CRC	
*HTR2A*	Serotonin receptor	CRC	--
*SCN7A/SCN5A/SCN2A*	Voltage-gated sodium channel proteins	CRC	Sodium channel blocker
*IKZF1*	Transcriptional factor, chromatin remodeling, hematopoietic cell differentiation	CRC	--
*PDZRN4*	Expressed in normal colon and nerves, tumor suppressor in liver cancer	CRC	--
*PAXIP1*	DNA repair, tumor suppressor	CRC	--
*XRCC4*	DNA repair, tumor suppressor	CRC	--
*RAP1GDS1*	Rap1 GTPase-GDP Dissociation Stimulator 1	Ovarian, peritoneal	--
*TET2*	Methylcytosine dioxygenase, myelopoiesis	Ovarian, peritoneal	--
*IL2*	Interleukin 2	Ovarian, peritoneal	--

## Data Availability

Not applicable.

## Abbreviations

BM (Brain Metastases); CDK (Cyclin-dependent Kinase); CNV (Copy Number Variant); CRC (Colorectal Cancer); CTCs (Circulating Tumor Cells); CTLA4 (Cytotoxic T-lymphocyte Antigen 4); DNA (Deoxyribonucleic Acid); EGFR (Epidermal Growth Factor Receptor); EM (Extracranial Metastasis); GABA (Gamma-aminobutyric Acid); HER2 (Human Epidermal Growth Factor Receptor 2); HLA-G (Human Leukocyte Antigen G); IGF-1 (Insulin-like Growth Factor); IL6 (Interleukin 6); LUAD (Lung Adenocarcinoma); NSCLC (Non-small Cell Lung Cancer); OXPHOS (Oxidative Phosphorylation); PI3K (Phosphatidylinositol-3-kinase); PLGF (Placental Growth Factor); PTEN (Phosphatase and Tensin Homolog); RBM (RNA-binding Motif); RNA (Ribose-nucleic Acid); SCLC (Small Cell Lung Cancer); SNV (Single Nucleotide Variant); SRS (Stereotactic Radiosurgery); TGFβ (Transforming Growth Factor Beta); TOLLIP (Toll Interacting Protein); WES (Whole-exome Sequencing).
